# Unveiling the rare: biliary tract ascariasis complicated by liver abscess and haemothorax

**DOI:** 10.1093/jscr/rjag780

**Published:** 2026-09-11

**Authors:** Ayesha Jamal, Rand AlSari, Shyma Haidar, Ayesha Shaikh, Husna Irfan Thalib, Omran Shrebaty, Sariya Khan, Carlos Enrique Garcia Franco, Raniah Rashed Alsubhi, Rothana Alnahari

**Affiliations:** General Medicine Practice Program, Batterjee Medical College, Prince Abdullah Al-Faisal Street, North Obhur, Jeddah 21442, Makkah Province, Saudi Arabia; General Medicine Practice Program, Batterjee Medical College, Prince Abdullah Al-Faisal Street, North Obhur, Jeddah 21442, Makkah Province, Saudi Arabia; General Medicine Practice Program, Batterjee Medical College, Prince Abdullah Al-Faisal Street, North Obhur, Jeddah 21442, Makkah Province, Saudi Arabia; General Medicine Practice Program, Batterjee Medical College, Prince Abdullah Al-Faisal Street, North Obhur, Jeddah 21442, Makkah Province, Saudi Arabia; General Medicine Practice Program, Batterjee Medical College, Prince Abdullah Al-Faisal Street, North Obhur, Jeddah 21442, Makkah Province, Saudi Arabia; College of Medicine, Sulaiman Alrajhi University, Mohammed Ali Al Suwailem Road, Al Bukayriyah 51941, Al-Qassim Province, Saudi Arabia; General Medicine Practice Program, Batterjee Medical College, Prince Abdullah Al-Faisal Street, North Obhur, Jeddah 21442, Makkah Province, Saudi Arabia; Department of Thoracic Surgery, King Abdullah Medical Complex, Prince Nayef Street, Al Shera'a District, Northern Obhur, Jeddah 23816, Makkah Province, Saudi Arabia; Department of Radiology, King Abdullah Medical Complex, Prince Nayef Street, Al Shera'a District, Northern Obhur, Jeddah 23816, Makkah Province, Saudi Arabia; Department of General Surgery, King Abdullah Medical Complex, Prince Nayef Street, Al Shera'a District, Northern Obhur, Jeddah 23816, Makkah Province, Saudi Arabia

**Keywords:** ascariasis, cholecystitis, liver abscess, hemothorax

## Abstract

Ascariasis, caused by *Ascaris lumbricoides*, is a common parasitic infection in developing countries, whereas biliary ascariasis is an uncommon manifestation that may present as cholangitis, biliary obstruction, or cholecystitis. We report the first documented case of biliary ascariasis in a 43-year-old Saudi man presenting with right upper quadrant pain and initially diagnosed with acute cholecystitis. Imaging revealed tubular filling defects within the biliary tree consistent with ascariasis, complicated by a liver abscess. The patient was treated with albendazole, followed by percutaneous drainage using a pigtail catheter and endoscopic retrograde cholangiopancreatography for worm extraction. Management was complicated by a haemothorax secondary to pigtail catheter insertion, requiring thoracic surgical intervention. The patient recovered successfully following multidisciplinary management. This case highlights the diagnostic challenges of biliary ascariasis in non-endemic settings, its potential for severe complications, and the importance of coordinated multidisciplinary care in achieving favorable outcomes.

## Introduction

Ascariasis, a helminthic infection caused by *Ascaris lumbricoides*, is the most common parasitic infestation of the gastrointestinal tract, affecting approximately one billion people worldwide [1]. Although predominantly found in tropical and subtropical regions, increasing global migration requires clinicians worldwide to recognize the clinical manifestations and management of biliary ascariasis [1, 2]. Transmission occurs through the fecal–oral route and commonly presents with abdominal discomfort and cholestatic symptoms [3]. Complications include anemia, gastrointestinal bleeding, intestinal obstruction, perforation, and, following migration into the biliary tree, obstructive jaundice [4]. We report the first case of a Saudi patient presenting with right upper quadrant (RUQ) pain who was diagnosed with common bile duct (CBD) ascariasis complicated by a liver abscess.

## Case presentation

A 43-year-old Saudi male with type 2 diabetes mellitus presented with a three-day history of sudden-onset RUQ pain radiating to both shoulders. The pain was constant, progressively worsening, aggravated by heavy meals, and unresponsive to analgesics. Associated symptoms included fever, anorexia, nausea, and dysuria. He denied vomiting, jaundice, changes in urine or stool color, previous similar episodes, prior surgery, or drug allergies.

On examination, he was hypotensive (90/67 mmHg), mildly tachycardic (96 bpm), and febrile (38.4°C), with improvement after intravenous fluids. Abdominal examination revealed epigastric and RUQ tenderness with a positive Murphy’s sign.

Laboratory investigations demonstrated leukocytosis (12.4 × 10^3^/μL) and serum lactate of 2 mmol/L. Liver function tests, direct bilirubin, amylase, and lipase were within normal limits. Chest and abdominal radiographs, electrocardiography, and cardiac markers were unremarkable. He was admitted under general surgery with a provisional diagnosis of acute cholecystitis.

**Figure 1:**
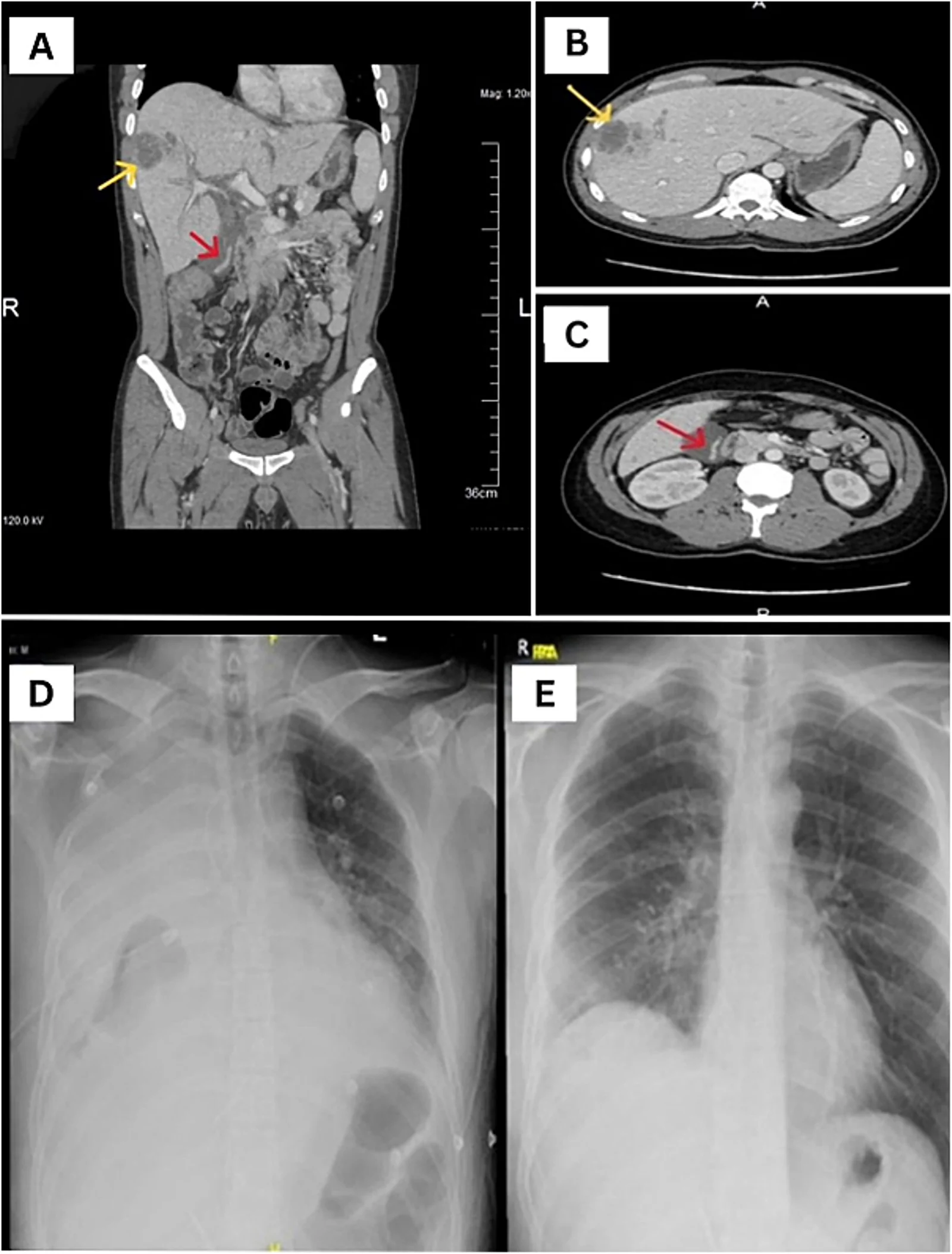
(A) CT scan of abdomen (coronary section) showing a multiloculated liver abscess in segment VIII, with tubular enhancing structures within the dilated gallbladder extending into the intrahepatic biliary tree. (B) CT scan (axial section) showing the multiloculated liver abscess. (C) CT scan (axial section) showing tubular enhancing structures within the gallbladder. (D) X-ray showing massive retained right-sided haemothorax with mediastinal shift. (E) X-ray done post-thoracotomy after removal of haemothorax showing clear lungs and trachea in centre, with visible pins from thoracotomy on right side.

Abdominal ultrasound showed mild hepatomegaly with diffuse fatty infiltration, a contracted gallbladder without stones, and a normal-caliber CBD. Contrast-enhanced computed tomography (CT) of the abdomen and pelvis revealed a multiloculated liver abscess in segment VIII measuring 3.9 × 2.6 × 4.1 cm, dilated intrahepatic bile ducts, and tubular enhancing structures extending from the gallbladder into the biliary tree, highly suggestive of biliary ascariasis complicated by liver abscess (Fig. 1A–C). Albendazole therapy was initiated.

The patient underwent ultrasound-guided aspiration of the liver abscess with pigtail catheter insertion. Planned endoscopic retrograde cholangiopancreatography (ERCP) was postponed after he developed acute dyspnea requiring oxygen therapy and intensive care admission. Following stabilization, ERCP successfully removed live *Ascaris* worms, and a plastic biliary stent was inserted.

Soon afterward, the patient’s respiratory condition deteriorated. Because he declined contrast administration, non-enhanced CT demonstrated bilateral pleural effusions, pulmonary consolidations, and free intraperitoneal fluid. Further imaging confirmed a massive right-sided haemothorax with mediastinal shift and right lung atelectasis secondary to the previously inserted pigtail catheter (Fig. 1D). A chest tube drained 500 mL of blood over 72 hours. Due to retained haemothorax, exploratory thoracotomy was performed. Large retained clots were evacuated without evidence of thoracic organ injury or active bleeding. The patient remained in the intensive care unit for 5 days. Chest tubes were removed on postoperative Days 2 and 4 (Fig. 1E). He recovered well and was discharged on Day 6 with a normal complete blood count, adequate pain control, and a scheduled outpatient follow-up.

## Discussion

Biliary ascariasis is an uncommon complication of *Ascaris lumbricoides* infection resulting from the migration of adult worms into the biliary tree. Patients commonly present with biliary colic, acute cholecystitis, cholangitis, pancreatitis, or obstructive jaundice, making diagnosis challenging because symptoms overlap with more common hepatobiliary diseases. Various presentations, management strategies, and outcomes of previously reported cases are summarized in Supplementary Material (Table S1).

Our patient’s presentation closely mimicked acute cholecystitis. Although ultrasonography has been reported as the preferred and most accurate initial diagnostic modality in most published cases, it was inconclusive in our patient. Contrast-enhanced CT established the diagnosis by demonstrating characteristic tubular structures within the gallbladder and biliary tree together with a multiloculated liver abscess (Fig. 1A–C). This highlights the value of CT when ultrasound findings are equivocal.

Management requires eradication of the parasite together with treatment of associated complications. Albendazole remains the standard antiparasitic therapy and has been successfully used in numerous reported cases. In our patient, medical therapy was combined with ultrasound-guided drainage of the liver abscess and ERCP for definitive removal of the worms. Prompt treatment is essential to relieve biliary obstruction, prevent recurrence, and reduce long-term complications.

A particularly unusual aspect of this case was the development of a massive retained haemothorax following pigtail catheter insertion for liver abscess drainage. Although pigtail catheters are generally considered safe, thoracic complications can be life-threatening. Similar cases reported in the literature have been managed using tube thoracostomy, insertion of additional thoracostomy tubes, image-guided drainage, fibrinolytic therapy, intrapleural streptokinase, video-assisted thoracoscopic surgery, or thoracotomy depending on severity [5]. In our patient, drainage with two thoracostomy tubes followed by exploratory thoracotomy successfully evacuated the retained haemothorax and resulted in complete recovery.

Ascariasis is uncommon among Saudi nationals and is reported predominantly among expatriate populations [6]. To date, only three published cases involving Filipino patients have been reported in Saudi Arabia [6–8]. To our knowledge, this is the first documented case in a Saudi patient. Furthermore, the coexistence of biliary ascariasis, liver abscess, and iatrogenic haemothorax following pigtail catheter insertion makes this presentation exceptionally rare. This case expands the regional literature and emphasizes maintaining a broad differential diagnosis for atypical hepatobiliary disease, even in low-endemic settings. It also highlights the importance of multidisciplinary management involving gastroenterologists, general surgeons, interventional radiologists, intensivists, and thoracic surgeons.

## Conclusion

This case highlights biliary ascariasis as a rare but important differential diagnosis in patients presenting with RUQ pain, even in regions where the disease is uncommon. Early recognition through appropriate imaging, timely antiparasitic therapy, image-guided drainage of associated liver abscesses, and ERCP are essential for successful management. Clinicians should also remain vigilant for rare but serious complications of invasive procedures, including haemothorax following pigtail catheter insertion, as prompt recognition and surgical intervention may be lifesaving.

## Supplementary Material

Supplementary_Material_-_Ascariasis_case_report_-_REVISED_rjag780

## Data Availability

All data supporting the findings of this case report are included within the article
