# Supplementary material for: Unveiling the rare: biliary tract ascariasis complicated by liver abscess and haemothorax
Source: J Surg Case Rep. 2026 Sep 11;2026(9):rjag780. doi: 10.1093/jscr/rjag780 (PMC13561878; doi:10.1093/jscr/rjag780)
Supplement: Supplementary file 1 [file supplementary_material_-_ascariasis_case_report_-_revised_rjag780.docx]

**Supplementary Material**

Table S1. Literature review summarizing key findings from similar previously reported cases.

**Literature Review**

Table 1. Literature review summarizing key findings from similar previously reported cases

| **Author- YOP** | **Patient** | **Presentation** | **Radiological and clinical findings** | **Complications** | **Management** |
| --- | --- | --- | --- | --- | --- |
| Abore KW et al. 2023 [1] | A 12-year-old male Ethiopian child | Colicky right upper quadrant pain, nausea, and vomiting for 3 days. | **US**: 1 cm dilation of CBD and intrahepatic duct.  2 ascaris worms within the biliary tree. | Obstructive jaundice secondary to intrabiliary ascariasis. | Patient was put on maintenance fluid and subsequently was dewormed with a 400 mg single dose of albendazole. |
| Asfaw YA et al. 2022 [2] | A 35-year-old woman | Intermittent, colicky, and non-radiating abdominal pain. | Abdominal examination revealed tenderness in right hypochondriac region.  **US**: worms in the distal CBD, with dilatation of  gall bladder and hepatic ducts. | Obstructive jaundice secondary to biliary ascariasis. | Intravenous saline, ceftriaxone, metronidazole and oral single dose was prescribed to eliminate worms. |
| Sarwar MS et al. 2022 [3] | A non-diabetic, non-alcoholic female | Vomiting mixed with worms since past one day and severe gastric pain for past 12 hours | **US**: revealed dilated CBD with 7 mm in diameter showing linear and peripheral tubular structures. | Cholangitis | ERCP and sphincterotomy was performed with dilation of CBD.  400 mg albendazole daily for three days. |
| Che Husin N et al. 2021 [4] | A 68-year-old woman | A dull aching epigastric pain and vomiting for one week. Yellowish discoloration of skin. | Tenderness in epigastric and right hypochondriac regions.  **US:** hyperechoic linear structures; worms in CBD which were causing biliary obstruction. | ascending cholangitis secondary to biliary ascariasis with history of symptomatic hepatolithiasis in right lobe of liver. | Management by endoscopic sphincterotomy and balloon trawling was done.  Intravenous fluids and 400 mg of oral albendazole for three days. |
| Rujeerapaiboon N et al. 2021 [5] | A 68 years old Thai man | Right upper quadrant pain, jaundice and fever | Mild Abdominal Distension.  **Cholangiography**: showed tubular filling structures in CBD and dilatation of the duct up to 7.3 mm diameter. | Hypotension and cholangitis | Endoscopic removal of Ascaris worms using balloon sweeping method.  400 mg albendazole once a day for 2 weeks. |
| Anzali BC et al. 2020 [6 | A 70-year-old woman | Presented with right upper quadrant (RUQ) pain with intermittent nausea and vomiting and a 3 kg weight loss during the last 2 months. | Epigastric and RUQ tenderness.  **US:** a distended GB with a parasitic lesion of 2.7 mm in diameter and also revealed a dilated CBD of 8 mm in diameter. | Presence of intrahepatic abscess | ERCP removal of ascaris roundworm and blood clots were extracted from the CBD.  100 mg mebendazole every 12 hours for 3 days along with 400 mg ciprofloxacin every 12 hours and 500 mg metronidazole every 8 hours, for a week. |
| Somani P et al. 2017 [7] | A 2-year-old Indian girl | Presented with symptoms of biliary colic and obstructive jaundice for 3 weeks. | **US**: revealed distended GB, dilation of intrahepatic biliary radicles, and a dilated CBD of 15mm.  Linear endoscopic US revealed dilated CBD with multiple hyperechoic structures; indicative of ascaris lumbricoides. | Dead Ascariasis | Removal of worms by multiple balloon sweeps on ERCP and patient was prescribed albendazole for deworming. |
| Pilankar, et al. 2003 [8] | A 35-year-old female | A dull aching epigastric pain and vomiting for 2 days. | **US**: revealed a dilated CBD and gallstones, and both US and CT showed findings of right and left intrahepatic stones along with an abscess in left lobe of liver. Biliary strictures with dilatation of intrahepatic biliary radicals were revealed on an ERCP. | Hepatolithiasis with ascariasis | Surgery was done to remove intrahepatic stones and segment 2 and 3 were resected due to its destruction by the abscess.  Oral antihelminth. |

US, Ultrasound; CBD, Common Bile Duct; RUQ, Right Upper Quadrant; ERCP, Endoscopic Retrograde Cholangiopancreatography; GB, Gall Bladder; CT, Computed Tomography.

**References**

1. Abore KW, Bacha T. Intrabiliary Ascariasis: A Case Report of an Uncommon Cause of Acute Abdomen and Obstructive Jaundice in Children. Int Med Case Rep J. 2023 Oct 1;16:689-92.
2. Asfaw YA, Asrat GT, Uddo TB, et al. Successful Management of Biliary Ascariasis in a High-Endemic Zone and Low-Resource Setting in Ethiopia. 2022 Dec 2;2022:1-4.
3. Sarwar MS, ALakkad A, Fawzy HA. Ascaris lumbricoides Infestation of Bile Ducts: Case Report. Asian J Res Med Pharm Sci. 2022 Nov 8;56-61.
4. Che Husin N, Mohamad IS, Ho KY, et al. Biliary ascariasis – A vicious cycle. Malays Fam Physician. 2021 May 27;16(2):83-5.
5. Rujeerapaiboon N, Kaewdech A. Massive biliary ascariasis: an unusual cause of acute cholangitis. BMJ Case Rep. 2021 Mar;14(3)
6. Anzali BC, Mohammadi N, Bahreini M, et al. Ascariasis in common bile duct resulting in a subhepatic abscess. PubMed. 2020 Oct 24;83(3):488-90.
7. Somani P, Sharma M, Jindal S. Endoscopic ultrasound appearance of dead Ascaris lumbricoides in the biliary tract. Endoscopy. 2017 Aug 10;49(11)
8. Pilankar K, Amarapurkar A, Joshi R, et al. Hepatolithiasis with biliary ascariasis – a case report. BMC Gastroenterol. 2003 Dec;3(1).
